# Plant Growth-Promoting Rhizobacteria Enhance Defense of Strawberry Plants Against Spider Mites

**DOI:** 10.3389/fpls.2021.783578

**Published:** 2022-01-06

**Authors:** Afsane Hosseini, Mojtaba Hosseini, Peter Schausberger

**Affiliations:** ^1^Department of Plant Protection, College of Agriculture, Ferdowsi University of Mashhad, Mashhad, Iran; ^2^Department of Behavioral and Cognitive Biology, University of Vienna, Vienna, Austria

**Keywords:** below-above ground interactions, plant growth-promoting rhizobacteria, plant growth, plant physiology, spider mite, life table, population dynamics

## Abstract

Plants mediate interactions between below- and above-ground microbial and animal communities. Microbial communities of the rhizosphere commonly include mutualistic symbionts such as mycorrhizal fungi, rhizobia and free-living plant growth-promoting rhizobacteria (PGPR) that may influence plant growth and/or its defense system against aboveground pathogens and herbivores. Here, we scrutinized the effects of three PGPR, *Azotobacter chroococcum*, *Azospirillum brasilense*, and *Pseudomonas brassicacearum*, on life history and population dynamics of two-spotted spider mites, *Tetranychus urticae*, feeding on aboveground tissue of strawberry plants, and examined associated plant growth and physiology parameters. Our experiments suggest that these three species of free-living rhizobacteria strengthen the constitutive, and/or induce the direct, anti-herbivore defense system of strawberry plants. All three bacterial species exerted adverse effects on life history and population dynamics of *T. urticae* and positive effects on flowering and physiology of whole strawberry plants. Spider mites, in each life stage and in total, needed longer time to develop on PGPR-treated plants and had lower immature survival rates than those fed on chemically fertilized and untreated plants. Reduced age-specific fecundity, longer developmental time and lower age-specific survival rates of mites feeding on rhizobacteria treated plants reduced their intrinsic rate of increase as compared to mites feeding on chemically fertilized and control plants. The mean abundance was lower in spider mite populations feeding on PGPR-treated strawberries than in those feeding on chemically fertilized and untreated plants. We argue that the three studied PGPR systemically strengthened and/or induced resistance in above-ground plant parts and enhanced the level of biochemical anti-herbivore defense. This was probably achieved by inducing or upregulating the production of secondary plant metabolites, such as phenolics, flavonoids and anthocyanins, which were previously shown to be involved in induced systemic resistance of strawberry plants. Overall, our study emphasizes that PGPR treatment can be a favorable strawberry plant cultivation measure because providing essential nutrients needed for proper plant growth and at the same time decreasing the life history performance and population growth of the notorious herbivorous pest *T. urticae*.

## Introduction

In terrestrial ecosystems, plants are the link between below- and above-ground communities, which may in consequence either indirectly, by altering plant traits, or directly, by moving up or down in the plant, interact with each other. During the last two decades, research on below-aboveground interactions has immensely intensified and many studies have focused on effects that cascade up and down between the communities of both subsystems (Van der Putten et al., 2001; Pineda et al., 2010; Hoffmann et al., 2011a,b; Friman et al., 2021b). Plant-mediated below-aboveground interactions involving micro-organisms of the rhizosphere may occur between plant-associated and/or free-living microorganisms (Bezemer and van Dam, 2005; Coskun et al., 2017; Dellagi et al., 2020), between micro-organisms and herbivores (Hoffmann et al., 2009; Patiño-Ruiz and Schausberger, 2014; Disi et al., 2019; Friman et al., 2021a) and, in turn, between micro-organisms and the herbivores’ natural enemies (Hoffmann et al., 2011a,b; Pineda et al., 2013; Gadhave et al., 2016). The rhizosphere microbial community may consist of a huge diversity of pathogenic, neutral and/or mutualistic micro-organisms. Among the usually mutualistic symbionts, it is especially mycorrhizal fungi, rhizobia and plant growth-promoting rhizobacteria (PGPR) that may influence aboveground plant growth and defense against pathogens and herbivores (Hoffmann et al., 2009, 2011a,b; Hoffmann and Schausberger, 2012; Khaitov et al., 2015; Pangesti et al., 2015; Nunes and Kotanen, 2017; Friman et al., 2021b). Here, we studied plant-mediated interactions between free-living soil bacteria that are commonly considered PGPR and aboveground herbivorous spider mites on strawberry plants.

Free-living soil bacteria such as *Azotobacter* spp., *Pseudomonas* spp., *Azospirillum* spp., and *Bacillus* spp. are well adapted to establish and thrive in competitive rhizosphere environments. Among other activities, these bacteria are well-known for using different kinds of inorganic nitrogen (N) from the soil and/or fixing atmospheric nitrogen (N_2_) (Steenhoudt and Vanderleyden, 2000; Dellagi et al., 2020), and thereby provide for nitrogen availability for uptake by the plants. Hence, free-living rhizobacteria can support plant growth and development via optimizing uptake of nitrogen and other nutrients such as phosphorus, iron and other micronutrients from the soil (Kempel et al., 2009; Gamalero and Glick, 2011). Due to the globally increasing demand for high agricultural productivity and the awareness of the disadvantages of using synthetic chemical pesticides and inorganic chemical fertilizers, ever growing efforts have been dedicated to sustainable agriculture (Gamalero and Glick, 2011). Application of biofertilizers such as PGPR to supply all or parts of nutritional requirements of crops is becoming more and more popular (Erturk and Çakmakçı, 2012; Conti et al., 2014; Andrade et al., 2019).

In addition to, and/or as a consequence of, promoting nutrient uptake by plants, PGPR may influence plant defense against below- and above-ground pathogens and herbivores. The phenomenon of belowground micro-organisms priming plants for defense against later occurring threats, or inducing a defense response against biotic stressors of aboveground plant parts, has been dubbed induced systemic resistance (ISR) (Dam, 2009; Glick, 2013; Pieterse et al., 2014). ISR is elicited by beneficial microbes in the rhizosphere and within-plants regulated by a network of endogenous signaling pathways, in which phytohormones play a major role (Pineda et al., 2010; Pieterse et al., 2014). Several studies have addressed indirect plant-mediated interactions between rhizobacteria and above-ground herbivores’ performance (Zehnder et al., 1997; Khan et al., 2018). These interactions are highly system- and context-specific and may range from positive (Khaitov et al., 2015) to neutral (Valenzuela-Soto et al., 2009) to negative (Zamioudis and Pieterse, 2012) effects on the herbivores, among others depending on the species of bacteria, plant and herbivore, the defensive array of the plant and the abiotic environmental conditions (Shah et al., 2021). Free-living rhizobacteria may increase the abundance of herbivores by enhancing the plant’s nutritional state but may simultaneously enhance growth and biomass of vegetative shoot and root tissue (Khaitov et al., 2015). Thus, rhizobacteria may affect plant tolerance to herbivore infestation by enhanced regrowth following herbivore injury (Disi et al., 2019). Free-living rhizobacteria can also trigger or enhance biosynthesis of defense-related chemical compounds in plants and, by this way, decrease life history performance and population growth of herbivores attacking aboveground plant tissue (Fahimi et al., 2014; Khoshfarman-Borji et al., 2020; Pourya et al., 2020).

Strawberry, *Fragaria* × *ananassa* Duchesne (Rosaceae), is one of the most frequently cultivated fruit crops around the world (Todeschini et al., 2018). Several studies documented that treatment of strawberry plants with PGPR may improve plant growth and reproduction (Martín et al., 2007; Pırlak and Köse, 2009; Roussos et al., 2012). Additionally, PGPR application may increase the concentration of some polyphenolic compounds in strawberry plants (Lingua et al., 2013; Todeschini et al., 2018), which may protect the plants against biotic stressors. However, the effects of PGPR on aboveground herbivores of strawberry have not yet been investigated. Two-spotted spider mites *Tetranychus urticae* Koch (Acari: Tetranychidae) are ubiquitous herbivores and highly destructive pests of a wide variety of agricultural and horticultural crops and may cause significant yield loss in field- and greenhouse-grown strawberry plants (Wermelinger et al., 1985; Migeon et al., 2010; Alizade et al., 2016). Severe spider mite infestations may strongly reduce strawberry fruit quality and quantity (Attia et al., 2013). While the effects of rhizosphere fungi on aboveground-feeding spider mite performance have been investigated in various plants such as bean (Hoffmann et al., 2009, 2011a; Hoffmann and Schausberger, 2012; Patiño-Ruiz and Schausberger, 2014), tomato (Pappas et al., 2018), and pepper (Pappas et al., 2021), it is unknown whether the presence of PGPR in the soil influences the defense system of strawberry plants against spider mites. This is an important question because spider mites usually thrive and are favored by high N-contents of plants (Wermelinger et al., 1985, 1991) such as presumably provided by PGPR (Khaitov et al., 2015 for common bean *Phaseolus vulgaris* L.).

Accordingly, we investigated whether treatment of strawberry plants with the PGPR *Azotobacter chroococcum* Beijerinck, *Azospirillum brasilense* Tarrand, Krieg & Döbereiner and *Pseudomonas brassicacearum* Achouak et al. positively affects the nutritional state of strawberry plants, which could either make them more prone to attack by two-spotted spider mites and enhance their performance and/or, additionally or alternatively, induce and/or strengthen the strawberry defense system against herbivory and thereby decrease the performance of spider mites. In detail, we scrutinized the effects of PGPR treatments on strawberry plant growth and physiology and the life history and population dynamics of *T. urticae* feeding on strawberry leaves and plants growing in substrates treated with PGPR, as compared to plants treated with chemical fertilizer and untreated plants (control).

## Materials and Methods

### Bacteria Preparation

The bacterial strains of *Azotobacter chroococcum* (Ac), *Azospirillum brasilense* (Ab), and *Pseudomonas brassicacearum* (Pb) used in the experiments were obtained from the Plant Pathology Laboratory, Department of Plant Protection, Faculty of Agriculture, Ferdowsi University of Mashhad, Iran. Bacteria were grown on nutrient agar (NA) for routine use, and maintained in nutrient broth (NB) with 15% glycerol at −80°C for long-term storage (Merck KGaA, Darmstadt, Germany). For each experiment, cultures were taken out from storage and grown on NA until use. A loop full of bacteria was transferred to 1-litre flasks containing NB, and grown aerobically in flasks on a rotating shaker with 150 rpm for 48 h at 24°C. The bacterial suspension was then diluted in sterile distilled water to a final concentration of 2 × 10^9^ CFUs (colony forming units) per ml, and the resulting suspensions were used to treat the strawberry plants (Zehnder et al., 1997). All three strains of the rhizobacteria species used in our study have previously revealed plant growth promoting effects on wheat (Mirzamohammadi et al., personal communication).

### Strawberry Culturing and Treatments

Seedlings of strawberry (*Fragaria* × *ananassa* cv. Selva) were periodically singly grown in 1-litre plastic pots (20 cm height × 17 cm diameter) filled with a substrate mixture of coconut peat (Kumari Coir Products, Singapore), clay and sand (1:1:1 by volume). The seedlings and pots were covered by transparent cylindrical fine-mesh cages (70 cm height × 30 cm diameter) and kept in a greenhouse at 26 ± 1°C, 65 ± 5% RH under natural lighting at the Agricultural Faculty, Ferdowsi University of Mashhad. The photoperiod was about 13 to 14/11 to 10 h L/D when the study was conducted in Mashhad, Khorasan-e-Razavi, Iran from early April to mid-May 2020.

We conducted two experiments on life history and population growth of *T. urticae* feeding on strawberry leaves and plants and one experiment on strawberry plant growth and physiology. The five treatments assigned to each experiment included (1) inoculation of strawberry roots with *A. chroococcum*, (2) with *P. brassicacearum*, and (3) with *A. brasilense* (no chemical fertilizer application for all three PGPR treatments) as well as (4) chemical fertilization (no PGPR application), and (5) control (no PGPR and chemical fertilizer application). For the PGPR treatments (1), (2), and (3), prior to planting, the roots of the seedlings were dipped in watery suspensions (density 2 × 10^9^ CFU/ml) of either Ac, or Ab, or Pb for 40 min. After that, the seedlings were air-dried for 30 min and then transplanted into the pots. The pots were tap-watered once every 2 days. For the chemical fertilization treatment, the recommended levels of nitrogen (N), potassium (K) and phosphorus (P) applied for conventionally grown strawberry plants were 170, 140, and 100 kg/ha (Hochmuth et al., 1996; Tabatabaei et al., 2006). Pots were fertilized three times per week with N and Ca (0.6 g/pot as calcium nitrate with total amount of 16.88 g/pot for 8 weeks), K and P (0.2 g/pot as di-potassium hydrogen phosphate with a total amount of 3.6 g/pot for 6 weeks) as well as Fe (0.1 g/pot as iron-chelate EDDHA with a total amount of 2.4 g/pot for 8 weeks) dissolved in 100 ml of distilled water.

### *Tetranychus urticae* Rearing

To initiate a strawberry-reared population of *T. urticae* (green form) specimens were collected from a population reared on whole common bean plants *Phaseolus vulgaris* kept under the above-described environmental conditions in the greenhouse. For rearing, strawberry plants (non-inoculated and fertilized) in the 3- to 4-leaf stage were periodically infested with mixed mobile stages of *T. urticae*, taken from other infested strawberry plants, and the plants covered by mesh cages (70 cm height × 30 cm diameter) to avoid any contamination by other herbivores. The strawberry-reared stock population was left to grow for at least four generations (∼45 days) before use in the experiments.

### Experiment 1. Life History and Life Table Analysis of *Tetranychus urticae*

This experiment was performed in a completely randomized design with leaves detached from 20 singly-potted strawberry plants, for each of the five treatments. About 1 month after seedling transplanting, leaves were clipped from clean strawberry plants that were in the 6- to 8-leaf stage and used to examine the life history of *T. urticae* in whole leaf tests (Alizade et al., 2016). To this end, the youngest fully developed leaf (average size 8.3 × 6.5 cm) of each strawberry plant was placed upside down on water-saturated cotton in a Petri dish (Ø 9 cm) and the Petri dishes were stored in a climate-controlled incubator at 25 ± 1.8°C, 70 ± 10% RH, and 16:8 h (L: D) photoperiod. Water was added daily to the Petri dishes to maintain cotton moisture. Every 3 to 4 days, the experimental mites were transferred to freshly detached leaves placed inside new Petri dishes.

To study the life history of *T. urticae*, an adult female/male pair was randomly withdrawn, using a fine camel-hair brush, from the strawberry-reared stock population, placed on a detached leaf from one of the five plant treatments and the female was allowed to lay eggs for 24 h. Subsequently, the adult mites and all but one egg were removed. Survival and developmental stage of each individual mite were checked twice daily at 10–12 h intervals until emergence of the adult. After reaching adulthood, a male from the same treatment was added to the female. If there were not enough males within the same treatment, additional males were collected from the strawberry-reared stock population and added to the leaf harboring the female; those males were not considered for life table analysis. Survival, longevity and fecundity of adult females and males were monitored once per day until natural death. The number of eggs laid by the females were daily counted and then removed. The number of replicates was 38, 40, 40, 45, 40 for Ac, Ab, Pb, chemical fertilizer and control, respectively.

From the survivorship, longevity and oviposition data of *T. urticae*, age-stage, two-sex life tables (Chi and Liu, 1985; Chi, 1988) were constructed using the computer program TWOSEX-MSChart (Chi, 2020). This program is freely available at http://140.120.197.173/Ecology/ (National Chung Hsing University, Taiwan) and http://nhsbig.inhs.uiuc.edu/wes/chi.html (Illinois Natural History Survey, IL, United States). Using this method, we calculated the age-stage specific survival rate (*S*_*xj*_), where x is the age and j is the stage; the age-stage specific fecundity (*f_x_*); the age-specific survival rate (*l*_*x*_); the age-specific fecundity (*m*_*x*_); the parameter *S*_*xj*_ represents the probability that an egg survives to age *x* and stage *j*; *l*_*x*_, the probability that an egg survives to age *x*; the net reproductive rate (*R*_0_), which is the mean number of offspring that a female mite can produce during her lifetime; the intrinsic rate of natural increase (*r*_*m*_) and the finite rate of increase (λ), which are the *per capita* rates of increase per time unit assuming unlimited and limited population growth; and the mean generation time (*T*), which is the average time required for the population to complete a full life cycle from egg to production of new eggs.

### Experiment 2. Strawberry Growth and Physiological Parameters

To characterize the effects of rhizobacteria treatments on strawberry growth and physiology, the number of flowers and leaves, leaf chlorophyll content and stomatal conductivity of the potted strawberry plants (6 weeks old, in the 6- to 8-leaf stage) were measured; these measurements were taken before releasing *T. urticae* to start the population dynamics experiment (experiment 3). To this end, the number of flowers and fully expanded trifoliate leaves (each composed of three leaflets) of the strawberry plants were counted, and the leaf chlorophyll content and stomatal conductivity were measured by a SPAD portable leaf chlorophyll meter (SPAD-502, Minolta Camera, Co., Japan) and a leaf porometer system (Sc-1), respectively. In both measurements, one newly expanded sunlit leaflet was randomly selected from each sampled plant. Each leaflet was one replication and measured once, and there were six replications for each treatment.

### Experiment 3. Population Dynamics of *Tetranychus urticae*

This experiment aimed at evaluating the effects of rhizobacteria and chemical fertilizer treatments as well as control treatment on the population dynamics of *T. urticae* on whole strawberry plants. To this end, a set of mixed *T. urticae* life stages including 3 adult females, 3 adult males and 3 protonymphs were gently transferred from the strawberry-reared stock population (reared on non-inoculated fertilized plants) to the abaxial surface of the youngest fully developed leaf of each potted strawberry plant (6 weeks old, in the 6- to 8-leaf stage) of each of the five treatments (8 replicate plants each per treatment). To make sure that the mites had successfully settled, we visually checked their fate after release. To prevent movement of *T. urticae* between pots, each pot was covered by an acrylic cylinder (70 × 30 cm) closed on top by an organdy-mesh and kept under the afore-mentioned environmental conditions of the greenhouse. Population development of *T. urticae* on the potted strawberry plants was assessed once per week by counting the number of mites on one strawberry leaf/plant; sampling started 1 week after mite infestation on 6 April and ended on 18 May 2020 for six consecutive samplings. On each sampling date, the youngest fully developed leaf of each strawberry plant was detached, placed into a labeled polyethylene bag, transferred to the laboratory and there all mite life stages (except eggs) were counted using a stereo microscope. The youngest fully developed leaf was chosen to standardize sampling among treatments. The detached leaves were subsequently returned to the experimental units they came from to let the mites reintegrate on their host plant.

### Statistical Analyses

For the data of experiment 1, the bootstrap technique, with 200,000 bootstrap samples, was used to estimate the means, variances, and standard errors of the life history parameters of *T. urticae* in each treatment. The bootstrap subroutine is included in the TWOSEX-MSChart (Chi, 2020) program. Paired bootstrap tests were used to compare the differences in *T. urticae* reproduction and life table among treatments (Mou et al., 2014) using TWOSEX-MSChart (Chi, 2020).

To assess the effect of rhizobacteria inoculation on growth and physiological measurements of strawberry plants (experiment 2), the data were analyzed by one-way ANOVA. Before analysis, the data were checked for normality and homogeneity of variance using Kolmogorov-Smirnov and Bartlett tests, respectively. Significant differences between treatment pairs were determined (*P* < 0.05) by *post hoc* Tukey’s test.

For the data of experiment 3, mite counting was repeated over time on the same strawberry plants; hence, the data were analyzed using repeated measures analysis of variance (RepANOVA), with sampling date as the repeated (within-subject) factor, and treatment being the between-subject factor. Statistical analyses of growth and physiological measurements of strawberry plants (experiment 2) and population dynamics of *T. urticae* (experiment 3) were performed using IBM SPSS 21 (IBM Corp, 2012).

## Results

### Experiment 1. Life History and Life Table Analysis of *Tetranychus urticae*

#### Immature Survival and Development, Adult Longevity and Reproduction

Development from egg to adult differed among the five treatments of strawberry (Table 1). Overall and for each life stage and total development, both females and males needed the shortest time on chemically fertilized plants and the longest on Ac-treated plants. In neither treatment, there was a significant difference in the developmental times of females and males except for longer total development of females than males reared on Ab-treated leaves (Table 1). The longest pre-oviposition period (POP) was observed in mites reared on Ac-treated plants. Fecundity was the highest on chemically fertilized plants and the lowest on Ac- and Ab-treated plants. Adult females and males reared on chemically fertilized plants lived the longest. In all treatments, adult longevity was significantly longer for females than for males (Table 1).

**TABLE 1:** Mean (± SE) developmental times of life stages and phases (in days) and fecundity (number of eggs/female) of *T. urticae* calculated using the age-stage, two-sex life table.

Variable		Treatment				
		Ac	Ab	Pb	CF	Control
Egg	♀	3.81 ± 0.10^A^	2.84 ± 0.10^B^	2.82 ± 0.11^B^	2.12 ± 0.06^D^	2.47 ± 0.09^C^
	♂	3.40 ± 0.33^A^	2.25 ± 0.25^C^	2.50 ± 0.55^B^	2.18 ± 0.12^C^	2.67 ± 0.21^B^
Larva	♀	2.06 ± 0.08^A^	1.88 ± 0.06^A^	1.97 ± 0.03^A^	1.06 ± 0.04^C^	1.19 ± 0.07^B^
	♂	2.11 ± 0.08^A^	2.00 ± 0.10^A^	2.10 ± 0.07^A^	1.00 ± 0.02^B^	1.17 ± 0.17^B^
Protonymph	♀	2.19 ± 0.08^A^	2.16 ± 0.07^A^	2.06 ± 0.04^A^	1.85 ± 0.07^C^	1.97 ± 0.05^BC^
	♂	2.20 ± 0.10^A^	1.91 ± 0.10^B^	2.17 ± 0.17^A^	1.89 ± 0.09^B^	2.17 ± 0.10^A^
Deutonymph	♀	2.75 ± 0.13^A^	2.53 ± 0.11^AB^	2.39 ± 0.08^B^	2.09 ± 0.06^C^	2.31 ± 0.08^B^
	♂	3.00 ± 0.58^A^	2.00 ± 0.00^D^	2.67 ± 0.21^B^	2.00 ± 0.13^D^	2.33 ± 0.21^C^
Total development	♀	10.81 ± 0.24^A^	9.41 ± 0.21^Ba^	9.24 ± 0.19^B^	7.12 ± 0.10^D^	7.94 ± 0.17^C^
	♂	10.77 ± 0.23^A^	8.16 ± 0.24^Cb^	9.44 ± 0.38*^B^*	7.07 ± 0.21^D^	8.34 ± 0.49^C^
POP^1^	♀	2.00 ± 0.14^A^	1.71 ± 0.11^AB^	1.52 ± 0.10^B^	1.44 ± 0.19^B^	1.56 ± 0.14^B^
Fecundity	♀	22.55 ± 2.50^D^	21.44 ± 2.73^D^	28.77 ± 2.71^C^	165.30 ± 7.00^A^	45.78 ± 3.90^B^
Adult longevity	♀	20.91 ± 0.72^Ba^	20.69 ± 1.10^Ba^	20.68 ± 0.60^Ba^	26.88 ± 0.80^Aa^	20.66 ± 0.58^Ba^
	♂	16.20 ± 1.53^Cb^	16.75 ± 0.75^Cb^	18.00 ± 1.57^Bb^	19.55 ± 0.72^Ab^	19.33 ± 1.02^Ab^

*Mites were reared on strawberry plants that received one of five treatments (Ac = Azotobacter chroococcum, Ab = Azospirillum brasilense, Pb = Pseudomonas brassicacearum, CF = chemical fertilizer, Control = without any soil treatment). ^1^POP = pre-oviposition period; different superscript uppercase letters indicate significant differences (P < 0.05) among treatments; different superscript lowercase letters indicate significant differences between males and females within each treatment (P < 0.05) based on paired bootstrap tests.*

#### Age-Specific Survivorship and Fecundity

**FIGURE 1:**
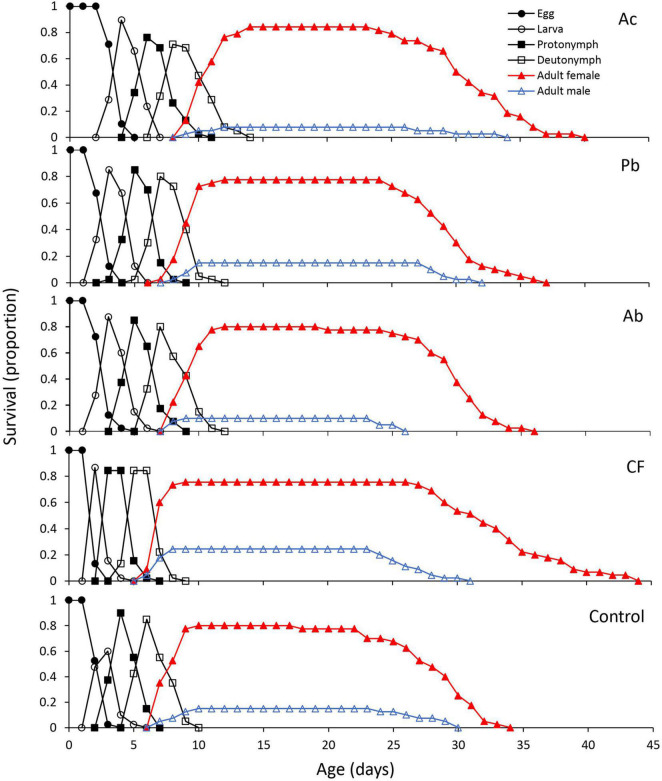
Age-stage specific survival curve (*s*_xj_) of *T. urticae* reared on leaves from strawberry plants subjected to one of five treatments, Ac (*Azotobacter chroococcum*), Ab (*Azospirillum brasilense*), Pb (*Pseudomonas brassicacearum*), CF (chemical fertilizer), and control (untreated).

The age-stage specific survival curves (*s*_xj_), i.e., the probability that a newborn will survive to age *x* and develop to stage *j*, of *T. urticae* reared on leaves from strawberry plants differed significantly among treatments (Figure 1). PGPR application to the substrate of strawberry plants decreased the immature survival rate (*s_a_*) in Ac, Ab and Pb treatments in comparison to the chemical fertilizer and control treatments (Figure 1). In all treatments, females survived longer than males. Females in the chemical fertilizer treatment survived longer than females in the other treatments. The overlapping curves of the different life stages result from varying developmental times of immature mites (Figure 1).

**FIGURE 2:**
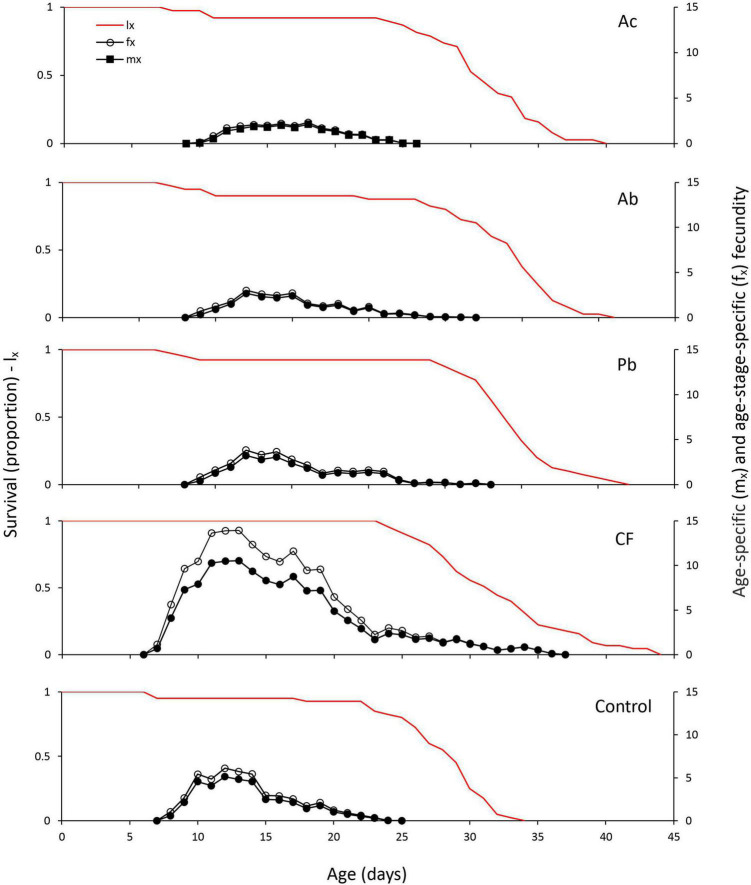
Age-specific survival (*l*_*x*_) and fecundity (*m*_*x*_, *f*_*x*_) curves of *T. urticae* reared on leaves of strawberry plants subjected to one of five treatments, Ac (*Azotobacter chroococcum*), Ab (*Azospirillum brasilense*), Pb (*Pseudomonas brassicacearum*), CF (chemical fertilizer), and control (untreated).

The age-specific survival rate (*l*_*x*_) represents the probability that an individual will survive to age *x* (Figure 2). Mites feeding on chemically fertilized plants had the longest survival times; no individual died before the age of 24 days. Also, mites reared on leaves of chemically fertilized strawberry plants started oviposition earlier (at 6 days age) than mites from the other four treatments. The age-specific fecundity (*m*_*x*_; mean number of eggs produced by each female at age x) and the age-stage specific fecundity (*f*_*x*_; mean number of eggs produced by each female at age x and stage j) curves show that the mean daily oviposition was pronouncedly higher in females feeding on leaves from chemically fertilized plants than in females from the other treatments; females feeding on chemically fertilized plants produced eggs over longer time than females in the other treatments (Figure 2).

#### Demographic Parameters

Demographic parameters of *T. urticae* differed among rhizobacteria-treated strawberry plants, and chemical fertilizer and control treatments of strawberry plants (Table 2). The highest intrinsic rate of increase (*r*_*m*_), net reproductive rate (*R*_0_) and finite rate of increase (λ) were observed in mites feeding on chemically fertilized plants and the lowest in mites feeding on Ac-treated plants. Mites reared on leaves of rhizobacteria-treated strawberry plants, especially those of Ac-treated plants, had longer mean generation times than mites feeding of chemically fertilized and control plants (Table 2).

**TABLE 2:** Mean (± SE) demographic parameters of *T. urticae* calculated using the age-stage, two-sex life table.

Parameter	Ac	Ab	Pb	CF	Control
*r*_*m*_	0.16 ± 0.01^d^	0.19 ± 0.01^cd^	0.20 ± 0.01^c^	0.35 ± 0.01*^a^*	0.26 ± 0.01^b^
*R*_0_	17.20 ± 2.46^c^	17.50 ± 2.31^c^	23.38 ± 2.80^c^	124.60 ± 11.70^a^	36.60 ± 4.20^b^
λ	1.18 ± 0.01^c^	1.20 ± 0.01^c^	1.20 ± 0.01^c^	1.42 ± 0.01^a^	1.30 ± 0.01^b^
*GT*	16.80 ± 0.31^a^	14.90 ± 0.20^b^	15.40 ± 0.28^b^	13.50 ± 0.23^c^	13.40 ± 0.20^c^

*Mites were reared on strawberry plants that received one of five treatments (Ac = Azotobacter chroococcum, Ab = Azospirillum brasilense, Pb = Pseudomonas brassicacearum, CF = chemical fertilizer, Control = without any soil treatment); r_m_ = intrinsic rate of increase (d^–1^), R_0_ = net reproductive rate (offspring/female), λ = finite rate of increase (d^–1^), and GT = generation time (d). Different superscript letters within rows indicate significant differences (P < 0.05) among treatments based on paired bootstrap tests.*

### Experiment 2. Strawberry Growth and Physiological Parameters

The number of flowers produced by young 6-week-old plants was significantly greater in Ac, Ab and Pb treatments than in the chemical fertilizer and control treatments (*F*_4,29_ = 7.5,*P* < 0.05;Table 3). The number of leaves did not differ among treatments (*F*_4,29_ = 0.70,*P* > 0.05). The highest leaf chlorophyll content was observed in Pb- and Ab-treated as well as chemically fertilized plants and the lowest in the Ac-treated and control plants (*F*_4,29_ = 17,*P* < 0.05). Stomatal conductivity was significantly enhanced in plants inoculated with rhizobacteria in comparison to chemically fertilized as well as control plants and was the greatest in the Pb treatment (*F*_4,29_ = 4.89,*P* < 0.05).

**TABLE 3:** Mean (± SE) growth parameters (*N* = 20 plants for flowers and leaflets) and physiological parameters (*N* = 6) of clean young strawberry plants (6 weeks after transplanting) that received one of five treatments (Ac = *Azotobacter chroococcum*, Ab = *Azospirillum brasilense*, Pb = *Pseudomonas brassicacearum*, CF = chemical fertilizer, Control = without any soil treatment).

Variable	Treatment				
	Ac	Ab	Pb	CF	Control
No. of flowers/plant	2.20 ± 0.20^a^	2.80 ± 0.22^a^	2.42 ± 0.20^a^	1.50 ± 0.50^c^	1.10 ± 0.16^c^
No. of leaflets/plant	17.66 ± 2.00^a^	14.75 ± 1.40^a^	16.00 ± 1.80^a^	18.75 ± 3.00^a^	14.25 ± 2.50^a^
Leaf chlorophyll content (μmol m^–2^)	34.39 ± 2.40^b^	42.70 ± 1.20^a^	43.22 ± 1.20^a^	41.27 ± 1.00^a^	29.23 ± 1.00^c^
Stomatal conductivity (mmol m^–2^ s^–1^)	15.47 ± 2.70^b^	15.32 ± 3.60^b^	21.82 ± 2.60^a^	11.00 ± 1.50^c^	9.72 ± 2.22^c^

*Different superscript letters within rows indicate significant differences among treatments based on ANOVA and post hoc Tukey’s tests (P < 0.05).*

### Experiment 3. Population Dynamics of *Tetranychus urticae*

**FIGURE 3:**
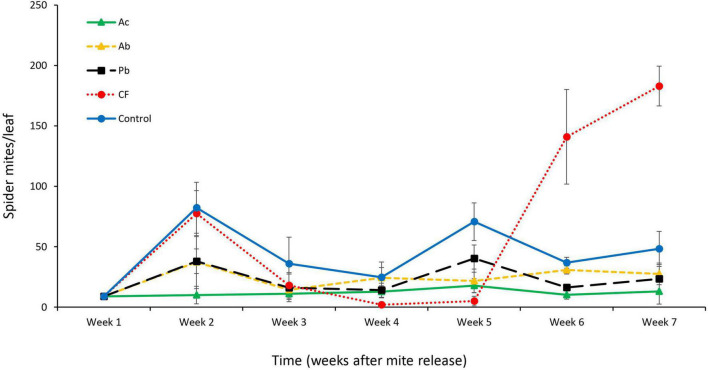
Mean (± SE) number of *T. urticae* (including all mobile developmental stages and adult males and females) per leaf at each weekly sampling interval (*n* = 6) on whole strawberry plants receiving one of five treatments, Ac (*Azotobacter chroococcum*), Ab (*Azospirillum brasilense*), Pb (*Pseudomonas brassicacearum*), CF (chemical fertilizer), and control (untreated).

In addition to significant main effects of time (six sampling dates) and treatment (three species of PGPR application, chemical fertilizer and control) on the abundance of *T. urticae*, also the interaction between time and treatment was statistically significant (RepANOVA: time: Greenhouse–Geisser adjusted *F*_5,75_ = 29.1, *P* < 0.01; treatment: Greenhouse–Geisser adjusted *F*_4,75_ = 56.9, *P* < 0.01; time by treatment: Greenhouse–Geisser adjusted *F*_20,75_ = 29.1, *P* < 0.01). In all treatments, a marked increase in spider mite abundance was observed 1 week after release (Figure 3); the population developing on the Ac-treated strawberries increased the least and had the lowest abundance; also the populations developing on the two other PGPR-treated plants (Ab and Pb) grew less than those on chemically fertilized and control plants (*F*_4,19_ = 12.7, *P* < 0.01; Figure 3). On chemically fertilized plants, the mite population dropped to very low levels in weeks 4 and 5, after an initial increase from week 1 to 2, but then steeply increased to 3- to 4-times higher abundances in weeks 6 and 7 than the populations in the other treatments (Figure 3). Overall, the population fluctuations of *T. urticae* were less pronounced on PGPR-treated plants than on chemically fertilized and control plants.

## Discussion

Below-ground beneficial micro-organisms may affect the performance of above-ground herbivores through plant-mediated mechanisms that may involve changes in plant physiology including enhancement of the plant’s nutritional state and/or induction of systemic resistance (ISR). The latter often results involves the production of secondary plant metabolites (Dean et al., 2009; Hoffmann et al., 2009; Kempel et al., 2009; Khaitov et al., 2015; Ruiu, 2020; Friman et al., 2021a). There exists ample evidence documenting that below-ground interactions with beneficial micro-organisms enhance nutrition and growth of strawberry plants (e.g., Pırlak and Köse, 2009; Todeschini et al., 2018; Andrade et al., 2019); however, the effects of these belowground interactions on life history performance and population dynamics of the cosmopolitan and most serious herbivorous pest of aboveground strawberry plant tissue, *T. urticae*, have not yet been studied. Here, we document adverse plant-mediated effects of three species of free-living rhizobacteria, *Azotobacter chroococcum* (Ac), *Azospirillum brasilense* (Ab) and *Pseudomonas brassicacearum* (Pb), on life history and population dynamics of *T. urticae* feeding on strawberry leaves and plants, and positive effects of these rhizobacteria on flowering and physiology of whole strawberry plants. Spider mites feeding on rhizobacteria-treated strawberry plants had longer developmental times and lower fecundity in comparison to those feeding on untreated and chemically fertilized plants. Overall, the intrinsic rate of increase (r_m_) of *T. urticae* was the lowest on Ac- and Ab-treated strawberry leaves and the highest on chemically fertilized strawberry leaves. Young rhizobacteria-treated clean plants produced more flowers than untreated and chemically fertilized plants but had similar numbers of leaves; stomatal conductivity tended to be higher in bacteria (especially Pb)-treated plants than in untreated and chemically fertilized plants whereas chlorophyll content was similar in Ac-treated and untreated plants and higher in chemically fertilized, and Ab- and Pb-treated plants.

Population dynamics of spider mites growing on PGPR-treated, chemically fertilized and untreated whole strawberry plants was in accordance with the adverse PGPR effects on spider mite life history. The mean abundance was lower in spider mite populations feeding on PGPR-treated strawberries than in those feeding on chemically fertilized and untreated plants. Time-dependent population development fluctuated similarly in all treatments except for the chemical fertilizer treatment. Mite abundance dropped to relatively low levels in the chemical fertilizer treatment in week 4 but grew exponentially from week 5 onward (corresponding to the 2nd and 3rd spider mite generation after transfer to the treated plants), which was expected considering the high intrinsic rate of increase. The drop in abundance in week 4 was probably caused by the higher nutrient content of leaf tissue of chemically fertilized plants making the mites staying longer on older leaves, and dispersing later to the newest leaf, than in the other treatments (weekly sampling concerned always the youngest fully developed leaf).

Beneficial below-ground microorganisms may influence the performance of herbivores via two major functional pathways, that is, enhancement of nutrition promoting plant growth and development, which in turn may favor the herbivores, or induced systemic resistance (ISR), which may disfavor the herbivores, or the combination thereof (Kempel et al., 2009; Pineda et al., 2010; Gamalero and Glick, 2011; Friman et al., 2021b). Many studies of above-belowground interactions showed that beneficial soil microorganisms such as free-living rhizobacteria can induce systemic resistance in above-ground plant parts and enhance the level of protection against herbivorous attackers (Zehnder et al., 1997; Koricheva et al., 2009; Khoshfarman-Borji et al., 2020; Ruiu, 2020; Friman et al., 2021a). Regarding interaction between the three species of rhizobacteria used in our study and aboveground herbivores, nothing has been known for *P. brassicacearum* (Pb). *A. brasilense* (Ab) had no effects on Hessian flies, *Mayetiola destructor*, in wheat (Prischmann-Voldseth et al., 2020) and soybean aphids *Aphis glycines* (Brunner et al., 2015) and positive effects on armyworm *Mythimna separata* in maize (Li et al., 2018). *A. chroococcum* (Ac) exerted positive effects on spider mites *T. urticae* in common bean (Khaitov et al., 2015) and both negative and positive effects (context- and plant genotype-dependent) on armyworm *M. separata* in maize (Li et al., 2018; Song et al., 2020).

In general, ISR may involve changes in the secondary metabolite profile such as alkaloids, phenols, terpenes, and flavonoids, some of which may inhibit feeding or digestion or have other toxic activity against herbivores (Pineda et al., 2010; Gadhave and Gange, 2013; Disi et al., 2019; Ruiu, 2020). For instance, reduced larval feeding and development of the beet armyworm *Spodoptera exigua* (Hübner) on *Bacillus* spp.-treated cotton plants was mediated by increased biosynthesis of gossypol, a phenolic aldehyde with insecticidal property, as compared to untreated plants (Zebelo et al., 2016). Our study revealed that both male and female spider mites, and in each life stage, needed longer time to develop on PGPR-treated strawberry plants and had lower immature survival rates than those fed on chemically fertilized and untreated plants. Reduced age-specific fecundity, longer developmental time and lower age-specific survival rates (as estimators of increased intrinsic rate of increase; Vehrsi et al., 1992) of the mites feeding on rhizobacteria-treated plants reduced their intrinsic rate of increase as compared to mites feeding on chemically fertilized and control plants. Collectively, these results suggest that rhizobacteria inoculation induces phytohormonal signaling (jasmonic acid – JA – and ethylene) that regulates the concentration of secondary plant metabolites (Stout et al., 2002; Pineda et al., 2010, 2013; Pieterse et al., 2014). For example, Steinite and Levinsh (2002, 2003) showed that induced resistance in strawberry, indicated by increased polyphenol oxidase, which is an important JA-inducible marker, negatively affected reproduction of *T. urticae*. Mouden et al. (2021) reported that induction of the JA pathway enhanced strawberry plant defense against *T. urticae* via increased production of phenolic acids, flavonoid compounds and anthocyanins. Similarly, Luczynski et al. (1990) observed that the development of *T. urticae* correlated negatively with foliar concentrations of phenolics. Therefore, we assume that in our study system increased biosynthesis of phenolic compounds and other secondary metabolites in rhizobacteria-treated strawberry plants acted as feeding deterrents, digestion inhibitors and/or toxins for the spider mites. Population dynamics of *T. cinnabarinus* Boisduval was inhibited in response to increased tannin levels (a polyphenol compound) in cotton (Ma et al., 2021). Similar to our study, Alizade et al. (2016) showed enhanced life history performance and population growth of *T. urticae* on N-fertilized strawberry plants. Increasing N content of the host plant tissue via chemical N fertilization improved the availability of dietary proteins, which typically benefits herbivores such as two-spotted spider mites (Alizade et al., 2016; Hosseini et al., 2018). Beneficial soil bacteria commonly also improve plant nutrition via increasing the uptake and concentration of a variety of nutrients, including nitrogen, and might also enhance the production of phytohormones that promote growth indicators like the rate of photosynthesis activity (Zhang et al., 2008; Pineda et al., 2010). In our study, application of rhizobacteria increased photosynthesis and growth compared to control plants, resulting in leaf chlorophyll content of Ab- and Pb-treated strawberry plants being similar to that of chemically fertilized plants and PGPR treatments having higher stomatal conductivity than chemically fertilized plants; while vegetative growth, indicated by the number of leaves, did not differ among treatments, reproductive growth, measured as the number of flowers, was significantly enhanced by rhizobacteria application, relative to chemically fertilized and untreated control plants.

Due to possible trade-offs between investment in growth and defense, the positive effects of mutualistic belowground microorganisms–plant interactions on plant growth do not necessarily and not always result in a net fitness benefit for the plant if the fitness of plant-associated herbivores is also enhanced and third trophic level natural enemies are left out (Gehring and Whitham, 2003). We did not assess the net effect of the rhizobacteria on plant growth at simultaneous defense against herbivores, because we just measured the plant characteristics of clean plants; however, our study documents that rhizobacteria can principally enhance both reproductive growth and defense of strawberry plants. Accordingly, we assume that rhizobacteria application may help attacked strawberry plants to limit the damage caused by spider mites, via inducing or increasing the production of secondary metabolites interfering with mite proliferation (Alizade et al., 2016), and to alleviate the deleterious effects of mite damage, via enhancing plant tolerance through vegetative or reproductive regeneration of herbivore-injured plant tissue, as a result of an improved nutritional state (McNaughton, 1983; Disi et al., 2019).

Overall, our results stress the importance of soil microbe-plant interactions on life history and population dynamics of aboveground herbivores. Rhizobacteria inoculation was more favorable than chemical fertilization in terms of decreasing the life history performance of *T. urticae* and limiting its population growth and improving reproductive growth of strawberry plants. Our study suggests that in crops with well-developed direct anti-herbivore defense systems, such as strawberry, PGPR can efficiently contribute to dampen herbivore infestation and provide for an improved nutritional state of the host plant. Future investigations should explore the consequences of PGPR application on aboveground tri-trophic interactions.

## Data Availability Statement

The raw data supporting the conclusions of this article will be made available by the authors, without undue reservation.

## Author Contributions

AH and MH conceived the study idea and designed the experiments. AH conducted the experiments, analyzed the data, and wrote the first draft of the manuscript. MH contributed to methods development and managed the project locally. PS gave advice on experimental design and contributed to results analysis and presentation. MH and PS contributed to writing and wrote sections of the manuscript. All authors contributed to manuscript revision, read, and approved the submitted version.

## Conflict of Interest

The authors declare that the research was conducted in the absence of any commercial or financial relationships that could be construed as a potential conflict of interest.

## Publisher’s Note

All claims expressed in this article are solely those of the authors and do not necessarily represent those of their affiliated organizations, or those of the publisher, the editors and the reviewers. Any product that may be evaluated in this article, or claim that may be made by its manufacturer, is not guaranteed or endorsed by the publisher.
